# Supplementary material for: Lateralizing Characteristics of Morphometric Changes to Hippocampus and Amygdala in Unilateral Temporal Lobe Epilepsy with Hippocampal Sclerosis
Source: Medicina (Kaunas). 2022 Mar 26;58(4):480. doi: 10.3390/medicina58040480 (PMC9029741; doi:10.3390/medicina58040480)
Supplement: Supplementary file 1 [file medicina-58-00480-s001.zip › medicina-1644887-supplementary.pdf]

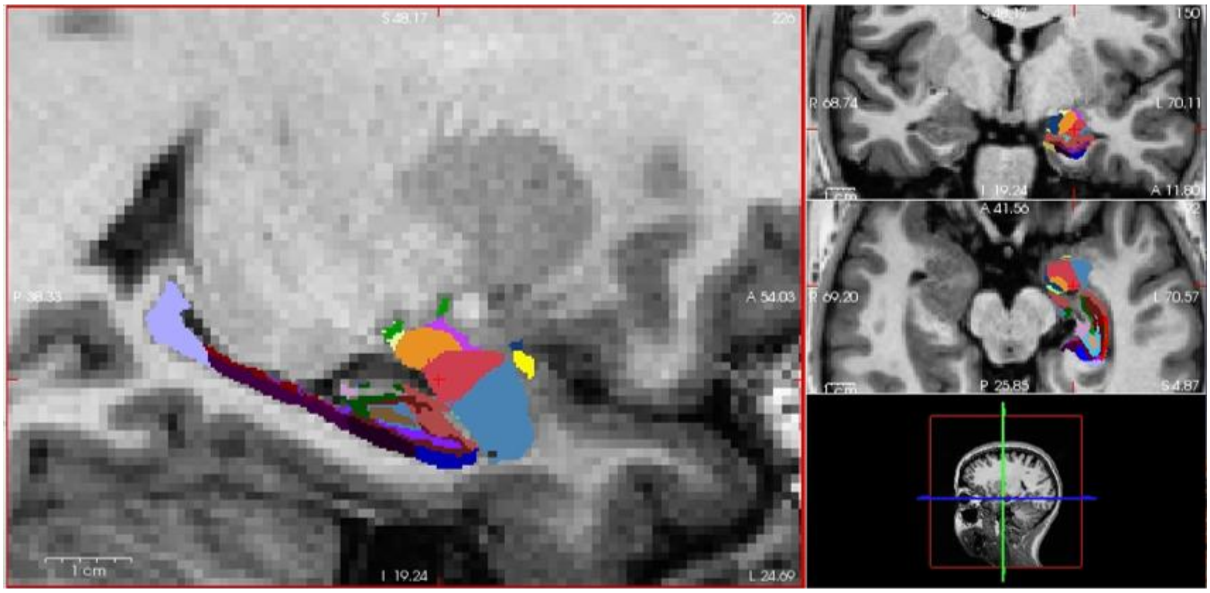

Supplementary Figure S1. Automated segmentation and cortical parcellation by FreeSurfer.

Supplementary Table S1. Comparison of subfield volumes (z-score) corrected for ICV in patients with LTLE and RTLE

Side	Region		LTLE	RTLE	p-value
Ipsilateral	Hippocampus	CA1 head	-3.2 ±0.7	-2.8 ±1.2	0.247[a]
		CA1	-3.1 ±0.8	-2.8 ±1.1	0.605[a]
		CA2/3 head	-2.3 ±0.9	-2.9 ±1.0	0.047[*]
		CA2/3	-2.2 ±0.8	-2.5 ±0.8	0.116
		CA4 head	-3.4 ±0.8	-3.2 ±1.2	0.367
		CA4	-3.4 ±0.9	-3.2 ±1.2	0.604
		DG head	-3.2 ±1.0	-3.2 ±1.3	0.992
		DG body	-3.0 ±0.8	-2.7 ±1.2	0.220
		SUB head	-2.9 ±0.8	-2.3 ±1.1	0.048[*]
		SUB body	-2.6 ±0.8	-2.8 ±1.3	0.388

	Amygdala	La	-1.6 ±1.1	-1.8 ±1.3	0.576
		Ba	-2.0 ±0.9	-1.6 ±1.3	0.482[a]
		AB	-2.0 ±0.8	-1.5 ±1.1	0.087
		Ce	-1.8 ±0.9	-1.9 ±0.8	0.565
		Me	-1.3 ±0.7	-0.9 ±0.8	0.153[a]
		Co	-2.1 ±1.0	-1.1 ±1.0	0.002*
		CAT	-1.7 ±0.8	-1.2 ±1.1	0.222[a]
Contralateral	Hippocampus	CA1 head	-0.8 ±0.9	-0.8 ±1.2	0.866
		CA1	-0.8 ±1.0	-0.6 ±1.1	0.517
		CA2/3 head	-0.6 ±1.0	-0.8 ±0.8	0.437
		CA2/3	-0.8 ±0.9	-0.6 ±0.8	0.522
		CA4 head	-0.7 ±1.0	-0.8 ±0.9	0.830
		CA4	-0.9 ±0.8	-0.8 ±0.9	0.791
		DG head	-0.6 ±0.9	-0.8 ±0.9	0.562[a]
		DG body	-1.0 ±0.7	-0.8 ±0.8	0.535
		SUB head	-2.9 ±0.8	-2.3 ±1.1	0.048*
		SUB body	-2.6 ±0.8	-2.8 ±1.3	0.388
	Amygdala	La	-1.0 ±0.8	-1.0 ±1.2	0.778
		Ba	-0.8 ±0.7	-1.3 ±1.1	0.092
		AB	-0.8 ±0.7	-1.2 ±1.0	0.136
		Ce	-1.1 ±0.7	-1.3 ±1.1	0.307
		Me	-0.5 ±0.7	-0.8 ±0.8	0.299
		Co	-0.7 ±0.7	-0.9 ±1.0	0.384
		CAT	-0.5 ±0.8	-1.1 ±0.9	0.013*

Data are mean ± standard-deviation values.

Analyzed by Independent t test

[a] Analyzed by Mann–Whitney test

* p-value < 0.05

LTLE, left temporal lobe epilepsy with hippocampal sclerosis, RTLE, right temporal lobe epilepsy with hippocampal sclerosis, CA, cornu ammonis; DG, granule cell layer of dentate gyrus; SUB, subiculum; La, Lateral nucleus; Ba, Basal nucleus; AB, Accessory Basal; Ce, Central; Me, Medial; Co, Cortical; CAT, Cortico-amygdaloid Transition Area
